# Safety and tolerability of a Muse cell-based product in neonatal hypoxic-ischemic encephalopathy with therapeutic hypothermia (SHIELD trial)

**DOI:** 10.1093/stcltm/szae071

**Published:** 2024-10-14

**Authors:** Yoshiaki Sato, Shinobu Shimizu, Kazuto Ueda, Toshihiko Suzuki, Sakiko Suzuki, Ryosuke Miura, Masahiko Ando, Kennosuke Tsuda, Osuke Iwata, Yukako Muramatsu, Hiroyuki Kidokoro, Akihiro Hirakawa, Masahiro Hayakawa, the SHEILD team, Xu Yue, Ryoko Goto, Takahiro Kanzawa, Yuki Hashimoto, Ryuichi Tanaka, Akinobu Taniguchi, Aiko Aoyama, Atsuna Kotani, Yoshihiro Tanahashi, Go Shoji, Akiko Saito, Miharu Ito, Kanji Muramatsu, Masatoshi Yoshikane, Tomoshige Tanimura, Koya Kawase, Taihei Tanaka, Kentaro Ueda, Seiji Hayashi, Takeshi Sahashi, Yuichiro Sugiyama, Azuma Ikari, Tetsuo Hattori, Yuichi Kato, Makoto Oshiro, Hiromasa Uchizono, Nao Matsuyama, Yumi Fujita, Yukihiro Suetake, Hisako Matsui-Hirai, Chiho Nishimura, Yasuko Watarai, Naoko Hayashi, Akemi Katayama, Yumiko Kobayashi, Fumie Kinoshita, Masaaki Mizuno, Toshimichi Yamamoto

**Affiliations:** Division of Neonatology, Center for Maternal-Neonatal Care, Nagoya University Hospital, Nagoya, Japan; Department of Advanced Medicine, Nagoya University Hospital, Nagoya, Japan; Division of Neonatology, Center for Maternal-Neonatal Care, Nagoya University Hospital, Nagoya, Japan; Division of Neonatology, Center for Maternal-Neonatal Care, Nagoya University Hospital, Nagoya, Japan; Division of Neonatology, Center for Maternal-Neonatal Care, Nagoya University Hospital, Nagoya, Japan; Division of Neonatology, Center for Maternal-Neonatal Care, Nagoya University Hospital, Nagoya, Japan; Department of Advanced Medicine, Nagoya University Hospital, Nagoya, Japan; Department of Pediatrics and Neonatology, Nagoya City University Graduate School of Medical Sciences, Nagoya, Japan; Department of Pediatrics and Neonatology, Nagoya City University Graduate School of Medical Sciences, Nagoya, Japan; Department of Pediatrics, Nagoya University Graduate School of Medicine, Nagoya, Japan; Department of Pediatrics, Nagoya University Graduate School of Medicine, Nagoya, Japan; Department of Clinical Biostatistics, Graduate School of Medical and Dental Sciences, Tokyo Medical and Dental University, Tokyo, Japan; Division of Neonatology, Center for Maternal-Neonatal Care, Nagoya University Hospital, Nagoya, Japan; Division of Neonatology, Center for Maternal-Neonatal Care, Nagoya University Hospital, Nagoya, Japan; Division of Neonatology, Center for Maternal-Neonatal Care, Nagoya University Hospital, Nagoya, Japan; Division of Neonatology, Center for Maternal-Neonatal Care, Nagoya University Hospital, Nagoya, Japan; Division of Neonatology, Center for Maternal-Neonatal Care, Nagoya University Hospital, Nagoya, Japan; Division of Neonatology, Center for Maternal-Neonatal Care, Nagoya University Hospital, Nagoya, Japan; Division of Neonatology, Center for Maternal-Neonatal Care, Nagoya University Hospital, Nagoya, Japan; Division of Neonatology, Center for Maternal-Neonatal Care, Nagoya University Hospital, Nagoya, Japan; Division of Neonatology, Center for Maternal-Neonatal Care, Nagoya University Hospital, Nagoya, Japan; Division of Neonatology, Center for Maternal-Neonatal Care, Nagoya University Hospital, Nagoya, Japan; Division of Neonatology, Center for Maternal-Neonatal Care, Nagoya University Hospital, Nagoya, Japan; Division of Neonatology, Center for Maternal-Neonatal Care, Nagoya University Hospital, Nagoya, Japan; Division of Neonatology, Center for Maternal-Neonatal Care, Nagoya University Hospital, Nagoya, Japan; Department of Pediatrics, Nagoya City West Medical Center, Nagoya, Japan; Department of Pediatrics, Toyohashi Municipal Hospital, Aichi, Japan; Department of Pediatrics, Nagoya City West Medical Center, Nagoya, Japan; Department of Pediatrics, Nagoya City West Medical Center, Nagoya, Japan; Department of Pediatrics and Neonatology, Nagoya City University Graduate School of Medical Sciences, Nagoya, Japan; Department of Pediatrics, Japanese Red Cross Aichi Medical Center Nagoya Daini Hospital, Nagoya, Japan; Department of Pediatrics, Japanese Red Cross Aichi Medical Center Nagoya Daini Hospital, Nagoya, Japan; Department of Pediatrics, Japanese Red Cross Aichi Medical Center Nagoya Daini Hospital, Nagoya, Japan; Department of Pediatrics, Okazaki City Hospital, Okazaki, Aichi, Japan; Department of Pediatrics, Ichinomiya Municipal Hospital, Aichi, Japan; Department of Neonatology, Anjo Kosei Hospital, Anjo, Aichi, Japan; Department of Pediatrics, Japanese Red Cross Aichi Medical Center Nagoya Daiichi Hospital, Nagoya, Japan; Department of Neonatology, Anjo Kosei Hospital, Anjo, Aichi, Japan; Department of Neonatology, Anjo Kosei Hospital, Anjo, Aichi, Japan; Department of Neonatology, Anjo Kosei Hospital, Anjo, Aichi, Japan; Department of Pediatrics, Japanese Red Cross Aichi Medical Center Nagoya Daiichi Hospital, Nagoya, Japan; Department of Neonatology, NHO Mie Chuo Medical Center, Tsu, Mie, Japan; Department of Advanced Medicine, Nagoya University Hospital, Nagoya, Japan; Department of Advanced Medicine, Nagoya University Hospital, Nagoya, Japan; Department of Advanced Medicine, Nagoya University Hospital, Nagoya, Japan; Department of Advanced Medicine, Nagoya University Hospital, Nagoya, Japan; Department of Advanced Medicine, Nagoya University Hospital, Nagoya, Japan; Department of Advanced Medicine, Nagoya University Hospital, Nagoya, Japan; Department of Advanced Medicine, Nagoya University Hospital, Nagoya, Japan; Department of Advanced Medicine, Nagoya University Hospital, Nagoya, Japan; Department of Advanced Medicine, Nagoya University Hospital, Nagoya, Japan; Department of Advanced Medicine, Nagoya University Hospital, Nagoya, Japan; Department of Advanced Medicine, Nagoya University Hospital, Nagoya, Japan; Department of Legal Medicine and Bioethics, Graduate School of Medicine, Nagoya University, Nagoya, Japan

**Keywords:** hypoxic–ischemic encephalopathy, neonates, cerebral palsy, hypothermia, mesenchymal stem cell, Muse

## Abstract

Hypoxic–ischemic encephalopathy (HIE), associated with high mortality and neurological sequelae, lacks established treatment except therapeutic hypothermia. Clinical-grade multilineage-differentiating stress-enduring (Muse) cells (CL2020) demonstrated safety and efficacy in nonclinical HIE rat models, thereby leading to an investigator-initiated clinical trial to evaluate CL2020 safety and tolerability in neonatal HIE as a single-center open-label dose-escalation study with 9 neonates with moderate-to-severe HIE who received therapeutic hypothermia. Each patient received a single intravenous injection of CL2020 cells between 5 and 14 days of age. The low-dose (3 patients) and high-dose (6 patients) groups received 1.5 × 10^6^ and 1.5 × 10^7^ cells/dose, respectively. The occurrence of any adverse event within 12 weeks following CL2020 administration was the primary endpoint of this trial. No significant changes in physiological signs including heart rate, blood pressure, and oxygen saturation were observed during or after administration. The only adverse event that may be related to cell administration was a mild γ-glutamyltransferase level elevation in one neonate, which spontaneously resolved without any treatment. All patients enrolled in the trial survived, and normal developmental quotients (≥ 85) in all 3 domains of the Kyoto Scale of Psychological Development 2001 were observed in 67% of the patients in this trial. CL2020 administration was demonstrated to be safe and tolerable for neonates with HIE. Considering the small number of patients, a randomized controlled confirmatory study is warranted to verify these preliminary findings and evaluate the efficacy of this therapy.

## Graphical Abstract

**Graphical Abstract:**
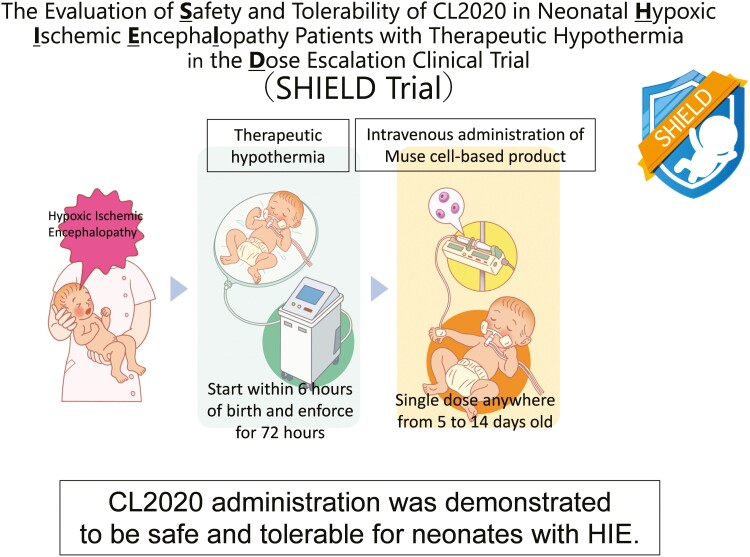

**Significance statement**

Muse cells are nontumorigenic, multipotent stem cells that are intrinsic to the body and function as repair stem cells. In this investigator-initiated clinical trial, 9 neonates with moderate-to-severe HIE who underwent therapeutic hypothermia received a single intravenous injection of a clinical-grade Muse cell product (CL2020). Serious adverse events that may be related to cell administration were not observed during the 18-month follow-up. Intravenous CL2020 administration was demonstrated to be safe and tolerable for neonates with HIE. It may lead to a new treatment for HIE by conducting a follow-up randomized controlled study to confirm the efficacy.

## Introduction

Neonatal hypoxic-ischemic encephalopathy (HIE) is an irreversible injury to the central nervous system caused by hypoxia and ischemia due to placental/umbilical blood flow blockage triggered by various causes, including premature separation of normally implanted placenta and umbilical cord prolapse and infection. HIE causes cerebral palsy, epilepsy, and delayed mental development. Approximately 30% and 60% of neonatal cerebral palsy cases in developed and developing countries, respectively, are related to HIE.^1^ Recent advances in perinatal care have dramatically improved the life expectancy of critically ill newborns; however, the incidence of cerebral palsy has not decreased and still occurs at a rate of 1-3 per 1000 live births.^2,3^

As the neurological disorders caused by HIE such as cerebral palsy and psychomotor developmental disorders are lifelong, the financial support and social burden for the patients and their supporting families are extremely high, with the economic cost per person estimated to be one million US dollars.^4^

Many drugs/treatments have been evaluated as novel therapies for HIE. However, no treatment has yet been proven effective for HIE, except for therapeutic hypothermia. The only treatment for HIE with a high level of clinical evidence is hypothermia, and its efficacy has been confirmed in several large randomized controlled trials.^5,6^ Although hypothermia, which aims to minimize the spread of injury due to ischemia and reperfusion injury, is strongly recommended as a treatment for neonates with moderate-to-severe HIE born at 36 or more weeks of gestational age, it currently fails to restore adequate function, and the number needed to treat (NNT) to reduce death or severe neurological sequelae at 18 months of age remains 9.^7^ Therefore, cell therapy/regenerative medicine has been recently applied to perinatal brain injury as a novel treatment modality.

Regarding stem cell therapy for HIE, preclinical studies have confirmed the efficacy of cord blood stem cells,^8,9^ and a phase I clinical trial was conducted.^10,11^ However, as these clinical trials used autologous cord blood stem cells, the availability of well-equipped facilities for cell preparation and human resources at the time of the birth of a neonate with HIE, which requires resuscitation, will markedly affect treatment feasibility. Therefore, we focused on multilineage-differentiating stress-enduring cells (Muse cells), a part of mesenchymal stem/stromal cells (MSCs), as an alternative that can be prepared in advance. Muse cells, which are intrinsic multipotent stem cells, possess an immunomodulatory system with human leukocyte antigen (HLA)-G,^12^ enabling direct administration without HLA-matching or immunosuppressants. Following intravenous administration, they migrate to damaged sites through the sphingosine-1-phosphate (S1P)-S1P receptor axis, self-renew, differentiate for tissue repair, and integrate into the host tissue microenvironment.^13^

We noted that the intravenous administration of human Muse cells in a rat model of HIE yielded positive outcomes.^14^ Muse cell administration resulted in amelioration in learning deficits and motor impairment. Notably, the Muse cells localized in the damaged areas of the brain and differentiated into neurons. These effects were more pronounced compared with those in MSCs that lacked the Muse cell subpopulation.

The main mechanism of MSC therapy is through trophic factors secreted by the cells.^15^ In addition to the effect of trophic factors, Muse cells have a better therapeutic effect because of the effects of migration and differentiation in the lesion site.^14^ Moreover, migration to the lesion site may enhance the therapeutic effect of the trophic factors. Our previous studies using other stem cells including bone marrow-derived MSCs with the rat model showed only minimal detection of these cells in the brain.^15,16^

Additionally, to assess the safety and effectiveness of a human allogenic Muse cell-based product called CL2020 (Life Science Institute, Inc., [LSII], Tokyo, Japan; nafimestrocel is the international nonproprietary name of cells in CL2020), we conducted experiments on HIE rat models. The results confirmed that CL2020 exhibited therapeutic effects without any observed toxicity.^17^

To further evaluate the safety and efficacy of CL2020, LSII conducted various clinical trials involving adult patients with different conditions, including acute myocardial infarction (JapicCTI-183834,^18^ JapicCTI-195067), stroke^19^ (JapicCTI-184103), epidermolysis bullosa^20^ (JapicCTI-184563), spinal cord injury (JapicCTI-194841), amyotrophic lateral sclerosis^21^ (jRCT2063200047), and acute respiratory distress syndrome associated with severe acute respiratory syndrome coronavirus 2 infection (jRCT2043210005).

However, the safety and tolerability of Muse cells in neonates remain unknown as they have never been administered to this population before. Therefore, to assess the safety and tolerability of CL2020 in patients with moderate-to-severe HIE who underwent hypothermia therapy, we have performed the first-in-neonate clinical trial: “The Evaluation of Safety and Tolerability of a multilineage-differentiating stress-enduring cell-based product cells in Neonatal Hypoxic-Ischemic Encephalopathy Patients with Therapeutic Hypothermia in the Dose Escalation Clinical Trial” (the SHIELD trial).^22^

## Methods

### Study design

**Figure 1.**
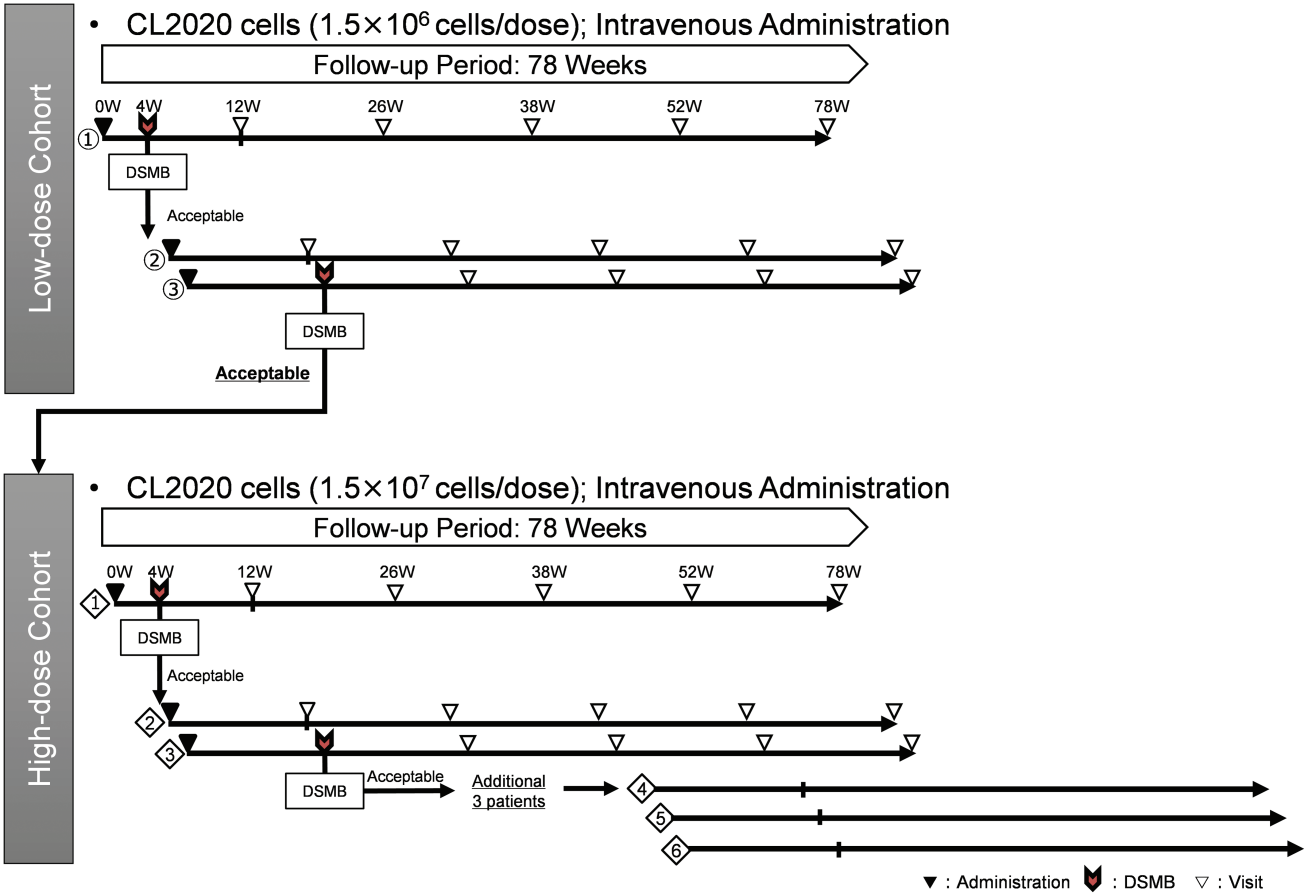
Study framework. The study used a 3 + 3 design, and the schematic diagram illustrates the framework of this clinical trial. It provides an overview of the enrolment schedule, the timing of CL2020 cell administration, as well as the assessments and visits for each patient. Additionally, it indicates when the Data Safety Monitoring Board (DSMB) meetings transpired. The DSMB convened for safety evaluation 4 weeks following CL2020 cell administration to the first patient in each cohort and 12 weeks following administration to the third patient in each cohort. The meetings aimed to determine if it was appropriate to proceed with enrolling the remaining participants on the basis of the safety data and evaluation.

This was a single-center open-label non-randomized dose-escalation exploratory clinical trial. To determine the optimal dosage of CL2020, we used a standard 3 + 3 dose-escalation design. The follow-up period extended until 78 weeks following CL2020 administration to each patient (Figure 1).

### Participants

Patients were recruited from Nagoya University Hospital or by receiving referrals from other hospitals in our district. The patients were registered for this trial after obtaining written informed consent from their legal parental authority, performing screening examinations, and verifying the eligibility on the basis of the inclusion and exclusion criteria shown before^22^ and as follows:

#### Inclusion criteria

The criteria were based on therapeutic hypothermia induction.^5,6^

1) Gestational age of at least 36 weeks, along with one of the following:

- Apgar score of ≤5 at 10 minutes after birth.
- Requirement of resuscitation (eg, tracheal intubation or positive pressure ventilation) for a minimum of 10 minutes.
- Blood gas pH level of <7.0 within 60 minutes of birth (from the umbilical cord blood, artery, vein, or peripheral capillary).
- Base deficit of ≥16 mmol/L in the blood gas within 60 minutes of birth (from the umbilical cord blood, artery, vein, or peripheral capillary).

2) Neonates assessed as moderate or severe encephalopathy (equivalent to stage 2 or more severe according to the Sarnat classification) at therapeutic hypothermia onset, specifically those exhibiting altered consciousness (somnolence, dullness, or coma) and one or more of the following symptoms:

- Hypotonia
- Absence of the “doll’s eye” reflex or abnormal reflexes, including abnormal pupillary response
- Reduced or absent sucking reflex
- Clinically evident seizures

1] Therapeutic hypothermia initiation within 6 hours of birth, maintained for approximately 72 hours continuously (documentation of therapy was reviewed at the time of enrolment)
2] Neonates with a birth weight of ≥1800 g
3] Heart rate (pulse) of ≥100 beats/minute and oxygen saturation (SpO_2_) level of ≥90% at enrolment
4] Neonates enrolled within the first 14 days of life. An adult guardian must provide a written informed consent as the legally authorized representative of the neonate during the consent process.
5] Consent must be voluntarily provided in writing by the legally authorized representative after being adequately informed about the study.

#### Exclusion criteria

1) Presence or suspicion of severe congenital abnormalities or chromosomal anomalies at the time of enrolment.
2) Scheduled for surgical procedures involving incisions (except for tracheostomy and gastrostomy) or planned radiation therapy within the first month after birth.
3) Planned administration of corticosteroid treatment for systemic effects for at least 5 consecutive days within the first month after birth.
4) Random blood glucose levels maintained at ≥200 mg/dL on at least 2 separate days by the time of enrolment.
5) Enrollment in another clinical trial (excluding observational studies).
6) Presence or suspicion of an active and severe infection at the time of the informed consent procedure.
7) Positive test results for hepatitis B surface antigen, hepatitis C virus antibody, human immunodeficiency virus antibody, human T-cell leukemia virus type 1 antibody, or positive syphilis serology (using various tests, including rapid plasma reagin, agglutination, slide flocculation, or automated testing).
8) History of severe hypersensitivity or anaphylactic reactions.
9) Presence of severe comorbidities unrelated to HIE, including significant liver, kidney, heart, lung diseases, hematologic disorders, or cerebral diseases (eg, congenital brain malformation).
10) Any other neonate deemed unsuitable for the study by the principal investigator or subinvestigator.

### Prohibited concomitant treatments and medications

The concomitant use of any of the following therapies and medications was not allowed from the informed consent procedure during the clinical trial period.

Corticosteroids at a dosage of 2 mg/kg/day or higher for >5 days, other human mesenchymal stem/stromal cell (MSC) products, processed cell products (excluding red blood cells), or other investigational drugs or products, and the use of investigational medical devices. In particular, corticosteroids can impact cell proliferation through RNA transcription,^23^ and we have considered the potential effects they may have on the functionality of the administered cells.

### Intervention

The CL2020, a clinical-grade product based on Muse cells (nafimestrocel), had been manufactured in the factory of the LSII. CL2020 cells are obtained from the bone marrow of healthy human donors and undergo expansion through adherent culture under hypoxic conditions to enhance the population of stage-specific embryonic antigen-3 (SSEA-3)-positive cells. The resulting preparation comprises Muse cells, with a purity of at least 50%, characterized by CD105 and SSEA-3 co-expression, along with a subset of MSC (CD105+; <50%). These cells are confirmed negative for the monocyte marker CD14, B-cell marker CD19, hematopoietic stem cell marker CD34, leukocyte marker CD45, and HLA-DR. Muse cells exhibit pluripotency and can secrete fibroblast, hepatocyte, and vascular endothelial growth factors. Additionally, they produce antifibrotic and fibrolytic factors, including matrix metalloproteinases.^24^

The CL2020 product comprised 1.5 × 10^7^ cells in a 15-mL preparation and was cryopreserved. After thawing the cells in a 37 °C water bath, they were removed using a syringe, centrifuged, redispersed in a 15-mL acetate Ringer’s solution, and filled the syringe. The solution was administered within 3 h after preparation, where the stability had been confirmed.

The dose volume of the CL2020 cells was 1.5 × 10^6^ cells (approximately 5 × 10^5^ cells/kg for 3 kg of body weight) dispersed in 1.5 mL of acetate Ringer’s solution for the low-dose cohort and 1.5 × 10^7^ cells (approximately 5 × 10^6^ cells/kg for 3 kg of body weight) dispersed in 15 mL of acetate Ringer’s solution for the high-dose cohort.

Following preparation of the investigational product, 1.5 mL of the product was intravenously administered for approximately 2 minutes to patients in the low-dose cohort and 15 mL for approximately 20 minutes to patients in the high-dose cohort (45 mL/h) using a syringe pump (TE-351Q, TERUMO, Tokyo, Japan). The prepared cells were intravenously administered once between 5 and 14 days after birth. Physiological signs (body temperature, diastolic and systolic blood pressures, and pulse [heart rate]) and oxygen saturation were monitored and recorded within 30 minutes before the initiation of the investigational product, within 10 minutes after the end of administration, and 1 h after the end of administration.

### Outcomes

The primary, secondary, and safety outcomes, and their statistical analysis, are presented below and in a previous publication^22^ Physiological signs and laboratory test values for safety assessment at specific points (Figure 1, Supplementary Table 1) were collected.

### Primary outcome

● Adverse events up to 12 weeks post-administration

Adverse events, defined as “any untoward medical occurrence not necessarily having a causal relationship with the treatment,” were identified regardless of their causal relationship to the Muse cell product administration. Adverse events were classified in accordance with the National Cancer Institute Common Terminology Criteria for Adverse Events ver. 5.0^25^ as follows:

Grade 1: mild; asymptomatic or mild symptoms; clinical or diagnostic observations only; no intervention indicated.

Grade 2: moderate; minimal, local, or noninvasive intervention indicated.

Grade 3: severe or medically significant but not immediately life-threatening; hospitalization or prolongation of hospitalization indicated.

Grade 4: life-threatening consequences; urgent intervention indicated.

Grade 5: death related to the adverse event.

Furthermore, serious adverse events were defined as follows^26^:

1) Results in death
2) Life-threatening
3) Requires inpatient hospitalization or causes prolongation of existing hospitalization
4) Results in persistent or significant disability/incapacity
5) May have caused a congenital anomaly/birth defect
6) Requires intervention to prevent permanent impairment or damage

### Secondary outcomes

1) Incidence of composite endpoints at 12, 26, 38, 52, and 78 weeks post-administration.

Evaluation of the presence/absence of the following events:

(i) Death
(ii) Ongoing need for respiratory support
(iii) Continued vasopressor or pulmonary vasodilator use

2) Mortality rates and overall survival

Evaluated at 78 weeks post-administration and throughout the trial period.

3) Duration of continuous respiratory support and vasopressor/pulmonary vasodilator use

Medications include dopamine, dobutamine, adrenaline, noradrenaline, milrinone, vasopressin, dl-isoprenaline hydrochloride, l-isoprenaline hydrochloride, nitric oxide, epoprostenol sodium, nitroglycerin, and alprostadil alfadex.

4) Infant development assessment

Using the Kyoto Scale of Psychological Development 2001 (KSPD)^27^ and Bayley-III^28^* at 78 weeks post-administration (*Values are for reference as they are adapted from standards in the US).

5) Developmental milestones at specified intervals

Evaluated at 26, 38, 52, and 78 weeks post-administration:

(i) Head control
(ii) Rolling over
(iii) Sitting position
(iv) Crawling
(v) Independent gait (52 and 78 weeks)
(vi) Meaningful words (52 and 78 weeks)

6) Spasticity incidence

Evaluated at 12, 26, 38, 52, and 78 weeks post-administration.

7) Epilepsy incidence

Evaluated from 2 to 78 weeks post-administration, defined by the International League Against Epilepsy.^29^

8) Magnetic resonance imaging (MRI) assessments

Performed immediately before and at 2 and 78 weeks post-administration and scored on the basis of a study by Barkovich et al.^30^ The detailed descriptions of the MR image protocol and MR imaging assessment are shown in the Supplementary method.

9) Gross Motor Function Classification System (Expanded and Revision) (GMFCS-E & R)^31^ Assessed at 78 weeks post-administration.

### Safety outcomes

1) Adverse events
2) Physiology signs, SpO_2_ levels, and laboratory values

The items to be investigated and the timing of the evaluation are listed in Supplementary Table 1.

### Data and Safety Monitoring Board

The Data and Safety Monitoring Board (DSMB) for this trial comprised 3 specialists in pediatric and perinatal care who were independent of the trial investigators. The DSMB meetings were held at predetermined intervals for both cohorts. Specifically, they transpired 4 weeks after the first patient’s administration and 12 weeks after the third patient’s administration in each cohort.

### Statistical analysis

All analyses in this study adhered to the intention-to-treat principle. Demographic data were summarized using descriptive statistics. Quantitative data were presented as means ± standard deviation. This exploratory clinical trial mainly aimed to confirm the safety and tolerability of the Muse cell product. Therefore, adverse events were analyzed in the safety analysis set, which included all participants enrolled in the study who received the investigational cell product. All adverse events were confirmed for the primary endpoint, regardless of whether they were related to the Muse cell product administration, and the proportions of adverse events along with their 95% confidence intervals were calculated using the Clopper–Pearson method.

Overall survival, defined as the time from birth to the date of death due to any cause, was assessed using the Kaplan-Meier method. Descriptive statistics for continuous variables and frequency and proportion for categorical variables were calculated for each secondary endpoint.

Statistical analysis was performed using SAS software (SAS Institute, version 9.4, Cary, NC, USA), and a *P*-value of <0.05 was considered statistically significant. Although certain endpoints including the provision of respiratory support and the use of vasoactive drugs may be influenced by pre-enrolment conditions, the potential baseline differences were not adjusted for in the analysis.

### Ethical approval

This study was approved by the Nagoya University Hospital Institutional Review Board (no. 312005) on November 13, 2019, and conducted in accordance with the Declaration of Helsinki and Good Clinical Practice.

## Results

During the time period available for enrollment in this study, a total of 11 referrals from other hospitals in our district were noted, and no eligible infants were born at Nagoya University Hospital. Of the 11 cases, one was excluded because the patient was in poor condition and could not be transported, and another case was excluded because the patient’s legal guardian could not provide consent owing to language barriers.

Nine patients’ legal parental authority provided written informed consent; all nine patients were implemented screening tests and registered for this trial. The DSMB meeting held when safety data were available for 3 participants in the low-dose cohort at 12 weeks after administration to the third patient determined that transfer to the high-dose cohort was possible. Therefore, 9 patients were finally enrolled in this study. All patients were successfully followed for 78 weeks without any instances of death or drop out, and the safety analysis set, full analysis set, and per protocol set included all 9 participants.

The patients’ background is shown in Table 1. Their gestational age and birth weight was 39.6 ± 1.5 weeks and 3308 ± 499 g, respectively. All cases were resuscitated for at least 10 minutes; the Apgar scores at 10 minutes were 5.6 ± 1.7 for all but one patient who had no record. Blood gas pH and base excess (BE) values within 60 minutes of birth were 7.05 ± 0.21 and − 15.78 ± 8.65, respectively. Neonatal seizures developed in 22.2% (2/9) of all patients.

**Table 1.** Patients’ characteristics.

			All cases (*N* = 9)	Low-dose cohort (*N* = 3)	High-dose cohort (*N* = 6)
Maternal characteristics	Age	year	34.1 ± 4.8	36.0 ± 2.6	33.2 ± 5.6
	Complications in pregnancy (total)		1	0	1
	Gestational diabetes		1	0	1
	Complications during labor and delivery (total)		5	2	3
	Premature separation of the normal placenta		1	1	0
	Shoulder dystocia		2	1	1
	Malrotation of the fetal head		1	0	1
	Others		1	0	1
	Concomitant medications		4	0	4
Sex		male	5	1	4
		female	4	2	2
Gestational age		weeks	39.56 ± 1.52	38.76 ± 2.36	39.95 ± 0.95
Birth weight		g	3307.6 ± 498.9	3328.0 ± 480.0	3297.3 ± 553.0
Birth length		cm	50.31 ± 3.10	49.67 ± 4.25	50.63 ± 2.79
Birth head circumference		cm	34.53 ± 1.38	33.93 ± 0.67	34.83 ± 1.60
Apgar score at 10 minutes			5.6 ± 1.7 (N = 8)	5.5 ± 2.1 (N = 2)	5.7 ± 1.8
Blood gas pH within 60 minutes of birth			7.05 ± 0.21	7.03 ± 0.30	7.06 ± 0.18
Blood gas BE within 60 minutes of birth			-15.78 ± 8.65	-13.37 ± 11.08	-16.98 ± 8.08
Sarnat classification on the date of birth		2 (moderate)	8	3	5
		3 (severe)	1	0	1
Neonatal seizure			2	1	1
Therapeutic hypothermia	Cooling methods	Selective head	1	0	1
		Whole body	8	3	5
	Starting time for cooling	hour	3.96 ± 1.47	3.79 ± 1.19	4.04 ± 1.69
	Cooling duration (excluding the rewarming period)	hour	74.09 ± 3.43	77.38 ± 4.68	72.45 ± 0.63
Age of cell administration		day	10.3 ± 2.5	8.7 ± 2.1	11.2 ± 2.5

Number of patients, mean ± SD.

Based on the Sarnat classification, 100.0% (3/3) of the low-dose cohort were classified as 2 (moderate), 83.3% (5/6) of the high-dose cohort as 2 (moderate), and 16.7% (1/6) as 3 (severe). One case complicated with parenchymal hemorrhages that was related to HIE was noted in the low-dose cohort.

Age at Muse cell product administration was 10.3 ± 2.5 days for all patients.

### Primary outcome

Adverse events until 12 weeks following Muse cell product administration are presented in Table 2. All adverse events were confirmed for the primary endpoint, regardless of whether it was related to the Muse cell product administration or not. The incidence and number of adverse events until 12 weeks following the administration were 100.0% (3/3) and 8 events and 100.0% (6/6) and 16 events for the low- and high-dose cohorts, respectively. One serious adverse event was noted, which was respiratory syncytial virus infection in one case in the high-dose cohort. However, only one adverse event that may be related to Muse cell administration was observed, including an increase in γ-glutamyltransferase (GTP) level in the low-dose cohort, which spontaneously resolved without any treatment.

**Table 2.** Primary outcome: adverse events up to 12 weeks.

SOC-PT	All cases (*N* = 9)	Low-dose cohort (*N* = 3)	High-dose cohort (*N* = 6)
All	24, 9	8, 3	16, 6
Gastrointestinal disorders	2, 2	1, 1	1, 1
Constipation	1, 1	1, 1	0, 0
Vomiting	1, 1	0, 0	1, 1
Hepatobiliary disorders	1, 1	1, 1	0, 0
Hepatic function abnormal	1, 1	1, 1	0, 0
Infectious and Infestations	1, 1	0, 0	1, 1
Respiratory syncytial virus infection	1, 1	0, 0	1, 1
Investigations	1, 1	1, 1	0, 0
Gamma-glutamyltransferase increased	1, 1	1, 1	0, 0
Metabolic and nutritional disorders	1, 1	1, 1	0, 0
Metabolic acidosis	1, 1	1, 1	0, 0
Nervous system disorders	1, 1	0, 0	1, 1
Muscle spasticity	1, 1	0, 0	1, 1
Pregnancy, puerperium and perinatal conditions	4, 4	1, 1	3, 3
Jaundice neonatal	1, 1	1, 1	0, 0
Umbilical granuloma	3, 3	0, 0	3, 3
Skin and subcutaneous tissue disorders	13, 8	3, 2	10, 6
Dermatitis diaper	3, 3	0, 0	3, 3
Eczema	2, 2	1, 1	1, 1
Eczema infantile	5, 5	0, 0	5, 5
Erythema	1 1	0, 0	1, 1
Skin erosion	1, 1	1, 1	0, 0
Asteatosis	1, 1	1, 1	0, 0

Number of events, number of patients.

The adverse events are tabulated by System Organ Class (SOC) and Preferred Term (PT) using MedDRA/J version 22.1.

### Secondary outcomes

#### Incidence of composite endpoints

No deaths were observed until 78 weeks following the administration or during the study period. The mean duration of continuous respiratory management was 13.0 and 11.0 days in the low-dose and high-dose cohorts, respectively; no cases were treated with continuous hypertensive or pulmonary vasodilators.

#### Infant and toddler development tests

The scores of infant and toddler tests including the Kyoto Scale of Psychological Development 2001 (KSPD) and Bayley-III at 78 weeks after administration are shown in Table 3.

**Table 3.** The scores of infant and toddler tests and the number of patients with ≥70 and ≥85 on the KSPD and Bayley-III at 78 weeks (± 28 days) following administration.

Scale	Domain		All cases (*N* = 9)	Low-dose cohort (*N* = 3)	High-dose cohort (*N* = 6)
Kyoto Scale	Posture-Motor	Mean ± SD	89.3 ± 27.7	70.3 ± 42.7	98.8 ± 13.2
		≥70 (n)	8	2	6
		≥85 (n)	7	2	5
	Cognitive-Adaptive	Mean ± SD	84.2 ± 23.8	72.0 ± 39.7	90.3 ± 11.9
		≥70 (n)	8	2	6
		≥85 (n)	6	2	4
	Language-Social	Mean ± SD	90.7 ± 26.5	76.7 ± 42.4	97.7 ± 14.9
		≥70 (n)	8	2	6
		≥85 (n)	7	2	5
	DQ	Mean ± SD	86.2 ± 24.8	72.7 ± 40.9	93.0 ± 12.3
		≥70 (n)	8	2	6
		≥85 (n)	7	2	5
	All 3 domains ≥ 70 (n)		8	2	6
	All 3 domains ≥ 85 (n)		6	2	4
Bayley-III^*^	Cognition	Mean ± SD	89.4 ± 16.3	83.3 ± 24.7	92.5 ± 12.1
		≥70 (n)	8	2	6
		≥85 (n)	7	2	5
	Language	Mean ± SD	84.3 ± 17.1	81.3 ± 24.6	85.8 ± 14.7
		≥70 (n)	7	2	5
		≥85 (n)	4	2	2
	Motor	Mean ± SD	94.9 ± 21.1	84.3 ± 33.4	100.2 ± 12.9
		≥70 (n)	8	2	6
		≥85 (n)	7	2	5

^*^These values are for reference only because they are not standardized for Japanese, and those standardized for children in the US are adapted to Japanese in this trial.

The KSPD scores for all domains (developmental quotient [DQ]) were 86.2 ± 24.8, 72.7 ± 40.9, and 93.0 ± 12.3 for all patients, low-dose, and high-dose cohorts, respectively. The percentages of cases with an overall domain score of ≥ 70 were 88.9% (8/9), 66.7% (2/3), and 100.0% (6/6) in all patients, low-dose, and high-dose cohorts, respectively. The percentages of patients with an overall domain score of ≥ 85 were 77.8% (7/9), 66.7% (2/3), and 83.3% (5/6) for all patients, low-dose, and high-dose cohorts, respectively.

Posture–movement, cognitive–adaptation, and language–social scores in the KSPD were 89.3 ± 27.7, 84.2 ± 23.8, and 90.7 ± 26.5, respectively, for all patients.

The percentages of patients with scores of ≥70 in all 3 domains were 88.9% (8/9), 66.7% (2/3), and 100.0% (6/6) for all patients, low-dose, and high-dose cohorts, respectively. The percentages of patients with scores of ≥85 in all 3 domains were 66.7% (6/9), 66.7% (2/3), and 66.7% (4/6) in all patients, low-dose, and high-dose cohorts, respectively.

#### Postnatal development assessment

The percentages of patients with each developmental milestone at each time point are shown in Table 4.

**Table 4.** The number of patients with each developmental milestone at each time point following administration.

Milestones	time point (weeks)	All cases (N = 9)	Low dose cohort (N = 3)	High dose cohort (N = 6)
Hold his/her head up	26	8	2	6
	38	8	2	6
	52	8	2	6
	78	8	2	6
Rolling over	26	7	1	6
	38	8	2	6
	52	8	2	6
	78	9	3	6
Sitting	26	1	1	0
	38	8	2	6
	52	8	2	6
	78	8	2	6
Crawling	26	2	1	1
	38	5	1	4
	52	7	2	5
	78	8	2	6
Walking	52	3	0	3
	78	8	2	6
Meaningful words	52	5	2	3
	78	7	2	5

Number of patients.

Regarding independent gait, the rate remained 33.3% (3/9) after 52 weeks and 88.9% (8/9) after 78 weeks in all patients. Regarding saying several meaningful words, the rate remained 55.6% (5/9) after 52 weeks and 77.8% (7/9) after 78 weeks for all patients.

#### Presence of spasticity

Presence of spasticity at each time point is shown in Supplementary Table S2.

Spasticity was 11.1% (1/9), 22.2% (2/9), and 11.1% (1/9) at 12, 26, and 38 weeks, respectively, for all patients, and remained the same thereafter until 78 weeks.

#### Presence of epilepsy

Epilepsy developed in 11.1% (1/9) in all patients and 33.3% (1/3) in the low-dose cohort but 0.0% (0/6) in the high-dose cohort. The infant experienced a focal seizure characterized by head and eyes turning to the left accompanied by tonic posturing of her left limbs.

#### MR imaging

MR images were scanned before, 2 weeks, and 78 weeks after administration and scored on the basis of the study by Barkovich et al.^30^ The representative MR images are shown in the Supplementary Figure. The MR imaging scores are shown in Supplementary Table S3.

The summation scores before administration, after 2 weeks, and after 78 weeks were 1.4 ± 1.9, 1.2 ± 2.0, and 1.0 ± 1.7, respectively.

#### Gross Motor Function Classification System

The gross motor function was evaluated using the Gross Motor Function Classification System (GMFCS)-Expanded and Revised after 78 weeks of treatment.

In the low-dose cohort, 66.7% (2/3) and 33.3% (1/3) were in levels I–II and III–V, respectively. In the high-dose cohort, 100.0% (6/6) were in levels I–II.

### Safety outcomes

**Figure 2.**
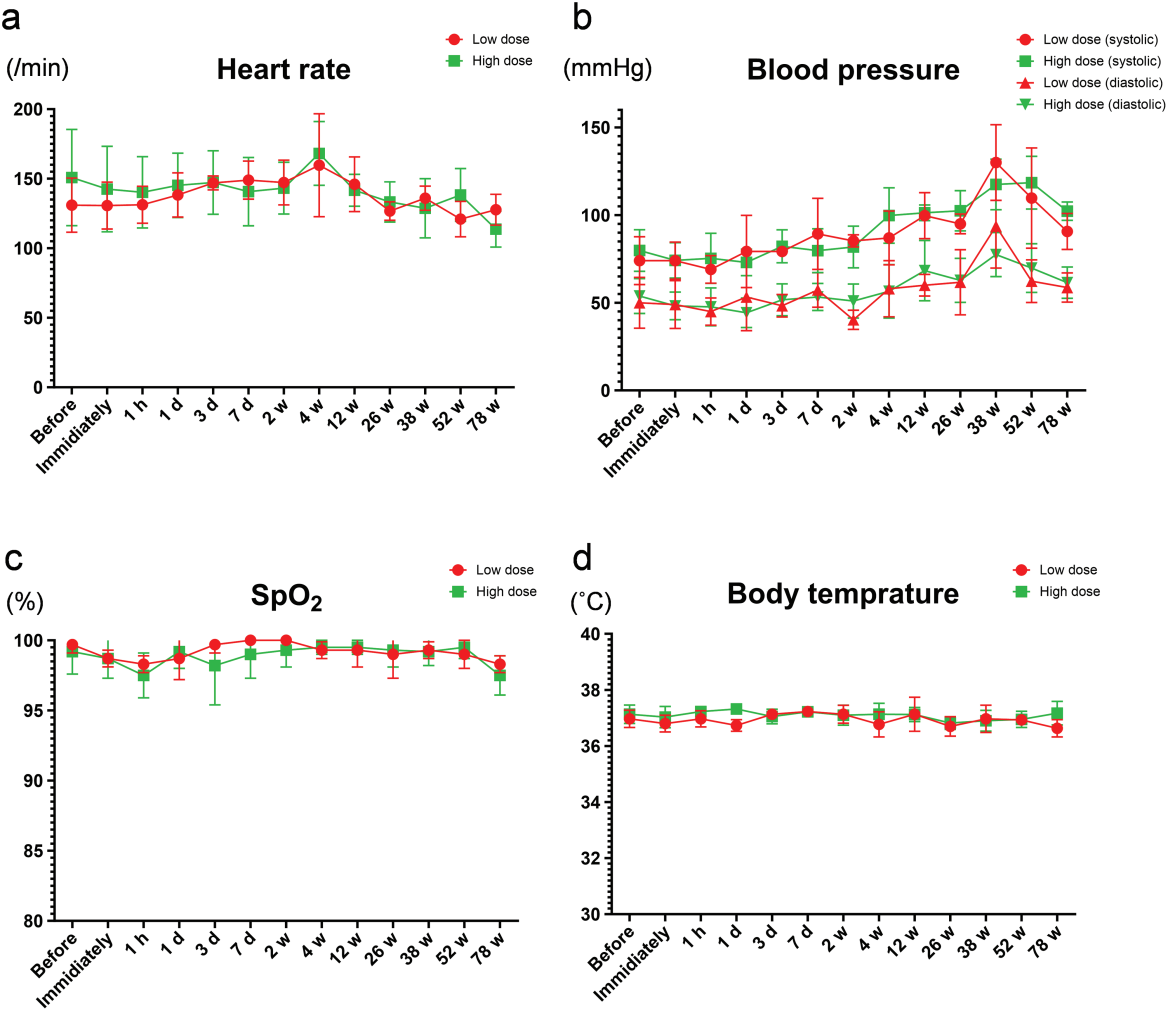
Physiological signs and SpO_2._ a) Heart rate, b) blood pressure, c) SpO_2_, d) Body temperature. These parameters are recorded before and immediately; at 1 hour; at 1, 3, and 7 days; and at 2, 4, 12, 26, 38, 52, and 78 weeks after CL2020 administration.

Physiological signs (systolic blood pressure, diastolic blood pressure, heart rate, and body temperature) and SpO_2_ levels at each time point after injection are shown in Figure 2. No abnormalities in physiological signs or SpO_2_ levels were observed.

Adverse events until 78 weeks following Muse cell product administration are shown in Table 5. The incidence and number of adverse events were 100.0% (3/3) and 30 events for the low-dose cohort and 100.0% (6/6) and 72 events for the high-dose cohort, respectively. However, none of them were associated with Muse cell product administration, except for the mildly elevated gamma GTP described above in the low-dose cohort.

**Table 5.** Adverse events up to 78 weeks.

SOC PT	All cases (*N* = 9)	Low-dose cohort (*N* = 3)	High-dose cohort (*N* = 6)
All	102, 9	30, 3	72, 6
Blood and lymphatic system disorders	2, 2	1, 1	1, 1
Anaemia	2, 2	1, 1	1, 1
Gastrointestinal disorders	14, 8	4, 3	10, 5
Angular cheilitis	1, 1	0, 0	1, 1
Constipation	4, 4	3, 3	1, 1
Diarrhoea	3, 1	0, 0	3, 1
Proctitis	1, 1	0, 0	1, 1
Umbilical hernia	1, 1	0, 0	1, 1
Vomiting	4, 4	1, 1	3, 3
General disorders and administration site conditions	3, 3	0, 0	3, 3
Pyrexia	3, 3	0, 0	3, 3
Hepatobiliary disorders	1, 1	1, 1	0, 0
Hepatic function abnormal	1, 1	1, 1	0, 0
Infectious and Infestations	37, 9	8, 3	29, 6
Exanthema subitum	1, 1	0, 0	1, 1
Gastroenteritis	1, 1	0, 0	1, 1
Hand-foot-and-mouth disease	2, 2	0, 0	2, 2
Nasopharyngitis	1, 1	0, 0	1, 1
Otitis media	1, 1	1, 1	0, 0
Pneumonia respiratory syncytial viral	1, 1	1, 1	0, 0
Rhinitis	5, 3	1, 1	4, 2
Upper respiratory tract infection	18, 7	2, 2	16, 5
Urinary tract infection	2, 1	2, 1	0, 0
Viral upper respiratory tract infection	1, 1	1, 1	0, 0
Corona virus infection	2, 2	0, 0	2, 2
Respiratory syncytial virus infection	2, 2	0, 0	2, 2
Injury, poisoning and procedural complications	2, 2	0, 0	2, 2
Arthropod sting	1, 1	0, 0	1, 1
Foot fracture	1, 1	0, 0	1, 1
Investigations	6, 3	1, 1	5, 2
C-reactive protein increased	1, 1	0, 0	1, 1
Fibrin D dimer increased	1, 1	0, 0	1, 1
Gamma-glutamyltransferase increased	2, 2	1, 1	1, 1
Platelet count increased	1, 1	0, 0	1, 1
Blood alkaline phosphatase increased	1, 1	0, 0	1, 1
Metabolic and nutritional disorders	1, 1	1, 1	0, 0
Metabolic acidosis	1, 1	1, 1	0, 0
Neoplasms benign, malignant and unspecified (incl cysts and polyps)	1, 1	0, 0	1, 1
Testicular yolk sac tumour^*^	1, 1	0, 0	1, 1
Nervous System Disorders	6, 2	4, 1	2, 1
Depressed level of consciousness	1, 1	0, 0	1, 1
Dyskinesia	1, 1	1, 1	0, 0
Epilepsy	1, 1	1, 1	0, 0
Muscle spasticity	2, 2	1, 1	1, 1
Seizure	1, 1	1, 1	0, 0
Pregnancy, puerperium, and perinatal conditions	4, 4	1, 1	3, 3
Jaundice neonatal	1, 1	1, 1	0, 0
Umbilical granuloma	3, 3	0, 0	3, 3
Skin and subcutaneous tissue disorders	22, 9	8, 3	14, 6
Dermatitis diaper	3, 3	0, 0	3, 3
Dry skin	1, 1	1, 1	0, 0
Eczema	7, 6	4, 3	3, 3
Eczema infantile	6, 6	0, 0	6, 6
Erythema	1, 1	0, 0	1, 1
Skin erosion	1, 1	1, 1	0, 0
Gianotti-Crosti syndrome	1, 1	1, 1	0, 0
Perianal erythema	1, 1	0, 0	1, 1
Asteatosis	1, 1	1, 1	0, 0

Number of events, number of patients.

The adverse events were tabulated by the System Organ Class (SOC) and Preferred Term (PT) using MedDRA/J version 22.1.

^*^As the genotypes of short tandem repeats at 15 loci in the oral mucosa and testicular tumor specimens matched at all loci, along with both specimens were male in the gender determination marker (amelogenin), both specimens were genetically derived from the same person, and a causal relationship with Muse cell-based product administration was denied.

Cardiotonic agents, antiepileptic drugs, hepatoprotective agents, antibiotics, cold medications, antipyretics, oral/inhaled medications for asthma, topical ointments, probiotics, laxatives, and iron supplements for anemia were the concomitant medications during the study period. However, none of these medications were administered with respect to any causative adverse events. Additionally, none of the abovementioned prohibited substances were used.

## Discussion

This study primarily aimed to evaluate the safety and tolerability of CL2020, a Muse cell-derived product, in neonates with HIE. In addition, as a secondary outcome, efficacy including neurodevelopment was evaluated. The primary endpoint, adverse events until 12 weeks following CL2020 administration, showed no serious cell administration-related adverse events; considering that the 3 low-dose cohorts and 6 high-dose cohorts were completed as planned without problems, it is believed that the safety and tolerability of the treatment of the cell product for infants with HIE were substantially confirmed in this study.

This study primarily aimed to confirm the safety of CL2020 up to 12 weeks after administration. Only one adverse event that may be related to Muse cell administration was observed, a mild increase in the γ-GTP level, which resolved without treatment. Clinical trials using CL2020 to confirm safety in adult diseases have been conducted. Noda et al. administered CL2020 to 3 patients with acute myocardial infarction.^18^ None of them showed any adverse side effects, and no cell therapy-associated safety concerns were noted. Fujita et al. reported the results of a phase 1/2 open-label study for 5 adult patients with dystrophic epidermolysis bullosa. All cases showed mild or self-limiting adverse effects; however, one patient developed a side effect, which may be related to CL2020; paresthesia within 24 h after infusion was also noted, which resolved in 14 days.^20^ Niizuma et al. performed a randomized placebo-controlled trial of CL2020 in subacute ischemic stroke and showed no major CL2020-related safety issues.^19^ Furthermore, Yamashita et al suggested that there are no safety concerns in ALS patients with multiple doses of CL2020.^21^ Regarding the treatment of acute or subacute stages of HIE by intravenous administration of cells, autologous umbilical cord blood stem cells^10,11^ and allogeneic umbilical cord tissue-derived MSCs have been used in the clinical trial to date, and all have been reported to be safe.

Generally, when intravenously administered, cells are distributed mainly in the lungs immediately after administration.^15^ However, no change in respiratory condition or pathological evaluation in nonclinical study using a rat model was noted.^17^ Furthermore, in this study, no change in physiological signs including respiratory status was observed during and immediately after cell administration. Notably, the 3 referenced CL2020 clinical studies reported no respiratory disorders. These collective findings suggest that intravenous CL2020 administration was safe not only in adults but also in children and neonates.

This study evaluated the treatment effect of CL2020 on infants with HIE although lacked a control group but compared data with the National Growth Survey on Preschool Children in Japan.^32^ In this study, at 52 weeks old, crawling and meaningful words were reported in 77.8% (7/9) and 55.6% (5/9) of the patients, respectively, aligning with national survey figures (96.9% and 57.6%, respectively). At 78 weeks old, meaningful words and unaided walking were reported in 77.8% (7/9) and 88.9% (8/9) of the patients in this study, which is almost comparable to national survey figures (94.7% and 100%, respectively).

In addition, data for evaluating the treatment effect were compared with the Baby Cooling Registry of Japan, which is a national registry for therapeutic hypothermia of patients with HIE in Japan, assessing survival, respiratory support, meaningful words, and GMFCS score ≤2 in 493 patients (Supplementary Table 4). Patients in this trial outperformed the registry, with 100% survival and 89% GMFCS score ≤2 compared with 92% and 77% in the registry, respectively. Furthermore, assessing a normal score (DQ ≥85) in all KSPD domains with the 170 cases for which the data were available, 67% achieved it in this trial compared with 33% in the registry. These findings indicate that CL2020 treatment in infants with HIE aligns with normal development and demonstrates promising clinical outcomes. Comparisons with the Baby Cooling Registry highlight positive results in survival, functional outcomes, and developmental scores, reinforcing the potential efficacy of CL2020.

Previous studies also reinforced the efficacy of CL2020. In a randomized placebo-controlled trial for subacute ischemic stroke,^19^ the CL2020 group exhibited a 40% response rate (modified Rankin Scale ≤2) compared with 10% in the placebo group. Although with limited sample sizes and lacked controls, trials conducted on acute myocardial infarction^18^ and dystrophic epidermolysis bullosa^20^ indicated marked improvements in cardiac function and ulcer size, respectively. Overall, these findings support the potential therapeutic benefits of CL2020.

Some clinical studies had been conducted for the treatment in the acute or subacute phase for HIE.^10,11,33^ In these clinical trials, cells were administered during hypothermia therapy. One notable advantage in this study is the flexibility of administering the cells at a later time point. In a nonclinical study using CL2020 with a rat HIE model,^17^ the treatment effect was observed at even 7 days after injury. Thus, the protocol for this study involved administering CL2020 to human neonates between 5 and 14 days after birth. This approach allowed physicians and patients’ families ample time to make informed decisions and prepare for the treatment. Furthermore, patients could undergo hypothermia therapy in any hospital, facilitating an earlier initiation of cooling. After rewarming, patients could be transferred to the hospital to receive additional treatment.

This study had some limitations. First, the sample size was small considering that this was an exploratory study conducted to observe safety and tolerability. Therefore, drawing conclusions regarding efficacy was not possible. Second, the nature of the patient population was heterogeneous, as this study used the criteria based on the one for therapeutic hypothermia induction. Third, the efficacy was evaluated at 78 months as a secondary outcome in this study. In Japan, it is standard to perform neurological evaluation at 1.5 and 3 years old using the abovementioned KSPD, and the registry, which was used for data comparison in this study, has data of only 2 similar time points. Moreover, we considered that 1.5 years should be sufficient for evaluating safety and feasibility, which was the main purpose. Therefore, an evaluation at 1.5 years was selected; however, it may be more likely to underestimate neurological development. Currently, we are conducting a long-term follow-up study with the same cases in this study and will confirm the development in the study.

## Conclusion

These results indicate that CL2020 administration is safe and tolerable for neonates with HIE. A randomized controlled trial is necessary to confirm these preliminary results and assess the efficacy of this treatment considering the limited number of patients in this study.

## Supplementary material

Supplementary material is available at *Stem Cells Translational Medicine* online.

szae071_suppl_Supplementary_Tables

szae071_suppl_Supplementary_Methods

szae071_suppl_Supplementary_Figures

## Data Availability

The datasets generated and analyzed during this study will not be publicly available due to the confidentiality clause. This clinical trial is the first study for neonates to investigate the safety and dosage of Muse cell product in a small group for HIE conducted in a single center. Data on individual subjects obtained in this clinical trial will not be disclosed at this time for the protection of personal information, because the risk of “re-identification” is significantly when the number of patients is small.

## Trial registration

ClinicalTrials.gov: NCT04261335, registered on February 7, 2020, https://clinicaltrials.gov/ct2/show/NCT04261335. Japan Registry of Clinical Trials: jRCT2043190112, registered on February 6, 2020, https://jrct.niph.go.jp/latest-detail/jRCT2043190112.
