# Supplementary material for: Safety and tolerability of a Muse cell-based product in neonatal hypoxic-ischemic encephalopathy with therapeutic hypothermia (SHIELD trial)
Source: Stem Cells Transl Med. 2024 Oct 14;13(11):1053–66. doi: 10.1093/stcltm/szae071 (PMC11555474; doi:10.1093/stcltm/szae071)
Supplement: szae071_suppl_Supplementary_Tables [file szae071_suppl_supplementary_tables.pdf]

Day 0 (during, and after administration)

1) Physiology signs (systolic and diastolic blood pressures, pulse rate (heart rate), temperature), oxygen saturation

   within 30 minutes prior to the initiation of theCL2020 (or immediately prior to administration if multiple measurements are performed), within 10 minutes after the end of administration and 1 hour after the end of administration.
2) Adverse events
3) Post-dose reaction (the presence of any reaction should be checked by interview, visual examination at 24 hours post-dose, etc.)
4) Composite endpoints (mortality, continuation of continuous respiratory support and use of continuous vasopressor or pulmonary vasodilator)

Days 1, 3 and 7 (1 and 3 (± 1) days and 1 week (± 2 days) after administration)

1) Physiology signs (systolic and diastolic blood pressures, pulse rate (heart rate), temperature), oxygen saturation
2) Laboratory tests

(i)	Hematology	RBC counts, Hb level, Ht level, WBC count, differential WBC counts (basophils, eosinophils, neutrophils, lymphocytes and monocytes) and platelet count
(ii)	Blood biochemistry	BUN, Cr, LDH, AST, ALT, ALP, γ-GTP, total bilirubin, bilirubin direct, CK, CRP, sodium, potassium, calcium and phosphate and blood glucose level
(iii)	Blood gases	pH, pCO_2 and BE
(iv)	Blood coagulation	PT, PT-INR, APTT, fibrinogen and antithrombin III
(v)	Urinalysis (random urine/qualitative)	Urinary protein, occult blood, sugar and pH

RBC, red blood cell; Hb, hemoglobin; Ht, hematocrit; WBC, white blood cell; BUN, blood urea nitrogen; Cr, creatinine; LDH, lactate dehydrogenase; AST, aspartate aminotransferase; ALT, alanine aminotransferase; ALP, alkaline phosphatase; GTP, glutamyltranspeptidase; CK, creatine kinase; CRP, C-reactive protein; BE, base excess; PT, prothrombin time; PT-INR, prothrombin time-international normalized ratio; APTT, activated partial thromboplastin time

3) Adverse events
4) Composite endpoints (mortality, continuation of continuous respiratory support and use of continuous vasopressor or pulmonary vasodilator)

Day 14 (2 weeks (± 4 days) after administration)

1) Physiology signs (systolic and diastolic blood pressures, pulse rate (heart rate),

temperature), oxygen saturation

2) Laboratory tests

(i)	Hematology	RBC counts, Hb level, Ht level, WBC count, differential WBC counts (basophils, eosinophils, neutrophils, lymphocytes and monocytes) and platelet count
(ii)	Blood biochemistry	BUN, Cr, LDH, AST, ALP, ALP, γ-GTP, total bilirubin, bilirubin direct, CK, CRP, sodium, potassium, calcium and phosphate
(iii)	Urinalysis (random urine/qualitative)	Urinary protein, occult blood, sugar and pH

3) Adverse events

4) Composite endpoints (mortality, continuation of continuous respiratory support and use of continuous vasopressor or pulmonary vasodilator)

5) Examination for epilepsy

6) MRI

Day 28 (4 weeks (± 7 days) after administration)

1) Physiology signs (systolic and diastolic blood pressures, pulse rate (heart rate), temperature), oxygen saturation

2) Laboratory tests

(i)	Hematology	RBC counts, Hb level, Ht level, WBC count, differential WBC counts (basophils, eosinophils, neutrophils, lymphocytes and monocytes) and platelet count
(ii)	Blood biochemistry	BUN, Cr, LDH, AST, ALT, ALP, γ-GTP, total bilirubin, bilirubin direct, CK, CRP, sodium, potassium, calcium and phosphate

3) Height, body weight and head circumference

4) Adverse events

5) Composite endpoints (mortality, continuation of continuous respiratory support and use of continuous vasopressor or pulmonary vasodilator)

6) Examination for epilepsy

Day 84 (12 weeks (± 14 days) after administration)

1) Physiology signs (systolic and diastolic blood pressures, pulse rate (heart rate), temperature), oxygen saturation

2) Laboratory tests

(i)	Hematology	RBC counts, Hb level, Ht level, WBC count, differential WBC counts (basophils, eosinophils, neutrophils, lymphocytes and monocytes)

		and platelet count
(ii)	Blood biochemistry	BUN, Cr, LDH, AST, ALT, ALP, γ-GTP, total bilirubin, bilirubin direct, CK, CRP, sodium, potassium, calcium and phosphate

3) Height, body weight and head circumference
4) Adverse events
5) Composite endpoints (mortality, continuation of continuous respiratory support and use of continuous vasopressor or pulmonary vasodilator)
6) Examination for spasticity
7) Examination for epilepsy

Day 182 (26 weeks (± 14 days) after administration)

1) Physiology signs (systolic and diastolic blood pressures, pulse rate (heart rate), temperature), oxygen saturation
2) Laboratory tests

(i)	Hematology	RBC counts, Hb level, Ht level, WBC count, differential WBC counts (basophils, eosinophils, neutrophils, lymphocytes and monocytes) and platelet count
(ii)	Blood biochemistry	BUN, Cr, LDH, AST, ALP, ALP, γ-GTP, total bilirubin, bilirubin direct, CK, CRP, sodium, potassium, calcium and phosphate

3) Height, body weight and head circumference
4) Adverse events
5) Composite endpoints (mortality, continuation of continuous respiratory support and use of continuous vasopressor or pulmonary vasodilator)
6) Examination for head control
7) Examination for rolling over
8) Examination for sitting position
9) Examination for crawling
10) Examination for spasticity
11) Examination for epilepsy

Day 266 (38 weeks (± 28 days) after administration)

1) Physiology signs (systolic and diastolic blood pressures, pulse rate (heart rate), temperature), oxygen saturation
2) Height, body weight and head circumference
3) Adverse events

4) Composite endpoints (mortality, continuation of continuous respiratory support and use of continuous vasopressor or pulmonary vasodilator)
5) Examination for head control
6) Examination for rolling over
7) Examination for sitting position
8) Examination for crawling
9) Examination for spasticity
10) Examination for epilepsy

Day 364 (52 weeks (± 28 days) after administration)

1) Physiology signs (systolic and diastolic blood pressures, pulse rate (heart rate), temperature), oxygen saturation
2) Laboratory tests

(i)	Hematology	RBC counts, Hb level, Ht level, WBC count, differential WBC counts (basophils, eosinophils, neutrophils, lymphocytes and monocytes) and platelet count
(ii)	Blood biochemistry	BUN, Cr, LDH, AST, ALP, ALP, γ-GTP, total bilirubin, bilirubin direct, CK, CRP, sodium, potassium, calcium and phosphate

3) Height, body weight and head circumference
4) Adverse events
5) Composite endpoints (mortality, continuation of continuous respiratory support and use of continuous vasopressor or pulmonary vasodilator)
6) Examination for head control
7) Examination for rolling over
8) Examination for sitting position
9) Examination for crawling
10) Examination for independent gait
11) Examination for meaningful words
12) Examination for spasticity
13) Examination for epilepsy

Day 546 (78 weeks (± 28 days) after administration)

1) Physiology signs (systolic and diastolic blood pressures, pulse rate (heart rate), temperature), oxygen saturation
2) Laboratory tests

(i)	Hematology	RBC counts, Hb level, Ht level, WBC count, differential WBC counts (basophils, eosinophils, neutrophils, lymphocytes and monocytes) and platelet count
(ii)	Blood biochemistry	BUN, Cr, LDH, AST, ALP, ALP, γ-GTP, total bilirubin, bilirubin direct, CK, CRP, sodium, potassium, calcium and phosphate

3) Height, body weight and head circumference
4) Adverse events
5) Composite endpoints (mortality, continuation of continuous respiratory support and use of continuous vasopressor or pulmonary vasodilator)
6) Examination for head control
7) Examination for rolling over
8) Examination for sitting position
9) Examination for crawling
10) Examination for independent gait
11) Examination for meaningful words
12) Examination for spasticity
13) Examination for epilepsy
14) Infant development face-to-face interview tests (KSPD and Bayley-III)
15) GMFCS-E&R
16) MRI

KSPD, Kyoto Scale of Psychological Development
GMFCS-E & R, Gross Motor Function Classification System (Expanded and Revision)

Supplemental Table 1. Observation, laboratory items and schedule

time point (weeks)	All cases (N = 9)	Low dose cohort (N = 3)	High dose cohort (N = 6)
12	1	0	1
26	2	1	1
38	1	1	0
52	1	1	0
78	1	1	0

Number of patients

Supplemental Table 2. Presence of spasticity at each time point following administration

	Time point (weeks)	All cases (N = 9)	Low dose cohort (N = 3)	High dose cohort (N = 6)
Basal ganglia (BG)	Pre	0.0 ± 0.0	0.0 ± 0.0	0.0 ± 0.0
	2	0.0 ± 0.0	0.0 ± 0.0	0.0 ± 0.0
	78	0.0 ± 0.0	0.0 ± 0.0	0.0 ± 0.0
Watershed (W)	Pre	1.4 ±1.9	3.0 ± 1.7	0.7 ±1.6
	2	1.2 ± 2.0	2.3 ± 2.5	0.7 ± 1.6
	78	1.0 ± 1.7	2.3 ± 2.5	0.3 ± 0.8
Basal ganglia/watershed (BG/W)	Pre	0.7 ± 1.4	1.3 ± 2.3	0.3 ± 0.8
	2	0.7 ± 1.4	1.3 ± 2.3	0.3 ± 0.8
	78	0.6 ± 1.3	1.3 ± 2.3	0.2 ± 0.4
Summation (S)	Pre	1.4 ± 1.9	3.0 ± 1.7	0.7 ± 1.6
	2	1.2 ± 2.0	2.3 ± 2.5	0.7 ± 1.6
	78	1.0 ± 1.7	2.3 ± 2.5	0.3 ± 0.8

Mean ± SD

Supplemental Table 3. Magnetic resonance imaging scores at each time point following administration

	SHIELD		Neonatal Research Network Japan
	Low dose	High dose	
Survival	100.0% (3/3)	100.0% (6/6)	91.5% (451/493)
Without respiratory support	100.0% (3/3)	100.0% (6/6)	90.0% (406/451)
Meaningful words	66.7% (2/3)	83.3% (5/6)	60.6% (261/431)
GMFCS ≤ 2	66.7% (2/3)	100.0% (6/6)	77.2% (348/451)
KSPD<br>≥ 85 in all three domains	66.7% (2/3)	66.7% (4/6)	33.1% (57/172)[※]<br>43.8% (57/130)[※※]

Number of patients

KSPD, Kyoto Scale of Psychological Development

[※] including 42 patients who died. [※※] excluding 42 patients who died

Based on data from 604 of the 756 patients who were treated with hypothermia between January 2012 and December 2016, excluding 152 patients for whom no data existed at 18 months, the following items (survival, no respiratory support, significant words, and GMFCS score of II or lower) were compared with 493 patients with the following condition: (i) births at 36 weeks or more of gestation, (ii) Sarnat classification at admission was two degrees or higher, and (iii) birth weight of 1,800 g or more. Furthermore, we compared the percentage of cases that were 85 or higher in all three domains, including “posture and movement,” “cognition and adaptation,” and “language and social” of the KSPD with the 172 cases for which the data were available.

Supplemental Table 4. Comparison with the Baby Cooling Registry of Japan
