# Supplementary material for: Safety and tolerability of a Muse cell-based product in neonatal hypoxic-ischemic encephalopathy with therapeutic hypothermia (SHIELD trial)
Source: Stem Cells Transl Med. 2024 Oct 14;13(11):1053–66. doi: 10.1093/stcltm/szae071 (PMC11555474; doi:10.1093/stcltm/szae071)
Supplement: szae071_suppl_Supplementary_Methods [file szae071_suppl_supplementary_methods.pdf]

Supplemental Method

MR Imaging Protocol

MR imaging was performed longitudinally before and after administration and at 18 months old using a 3T scanner (MAGNETOM Trio, a Tim System; Siemens, Erlangen, Germany) with a 32-channel phased-array head coil. During the neonatal period, all infants underwent imaging without sedation. At 18 months old, sedation was induced using oral chloral hydrate (80 mg/kg) before the examination. When patients were inadequately sedated following chloral hydrate intake, intravenous thiamylal sodium was administered (1–2 mg/kg). The following images were acquired for scoring: axial T1-weighted imaging (TR/TE, 1,800/2.1 ms; slice thickness, 1 mm) and axial T2-weighted imaging (TR/TE, 5000/126 ms; slice thickness, 2 mm) during the neonatal period; axial T1-weighted imaging (TR/TE, 400/11 ms; slice thickness, 5 mm); and axial T2-weighted imaging (TR/TE, 4000/70–80 ms; slice thickness, 5 mm) at 18 months old.

Assessment of MR Imaging

The MR imaging assessment was conducted through visual inspection by a pediatric neurologist (HK) who was blinded to clinical parameters, according to Barkovich's classification. The scores used included the basal ganglia (BG) score, watershed (W) score, and combined basal ganglia/watershed (BG/W) score, which was developed as a combination score to include the BG and W cortex patterns of injury and the summation (S) score, which was the arithmetic sum of the BG and W scores.
