# Supplementary material for: Safety and tolerability of a Muse cell-based product in neonatal hypoxic-ischemic encephalopathy with therapeutic hypothermia (SHIELD trial)
Source: Stem Cells Transl Med. 2024 Oct 14;13(11):1053–66. doi: 10.1093/stcltm/szae071 (PMC11555474; doi:10.1093/stcltm/szae071)
Supplement: szae071_suppl_Supplementary_Figures [file szae071_suppl_supplementary_figures.pdf]

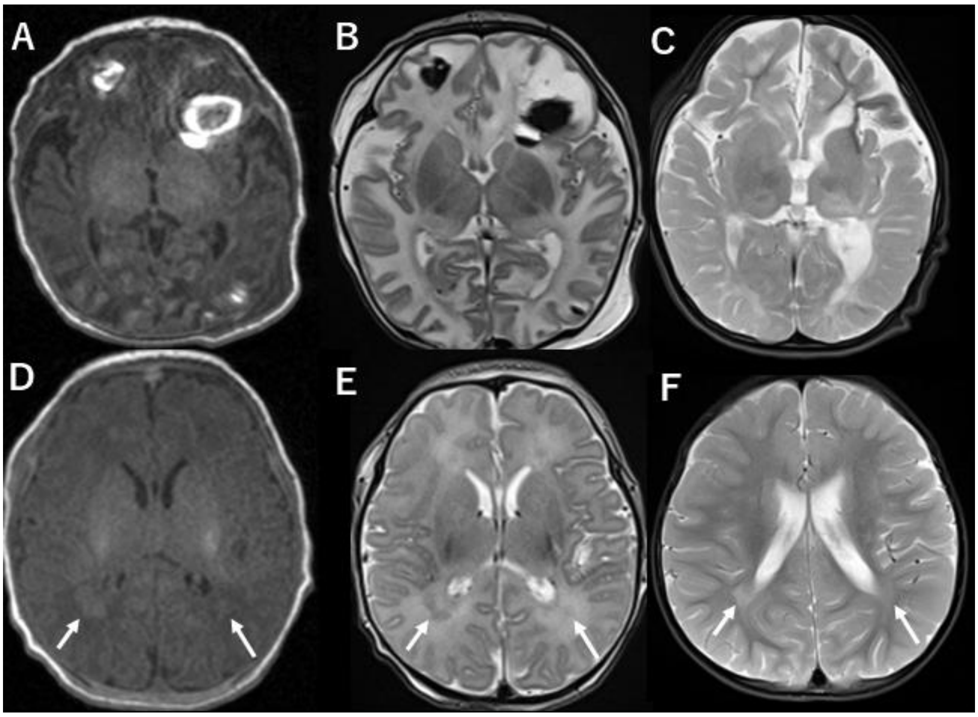

Supplemental Figure

A and **B** depict axial T1- and T2-weighted magnetic resonance (MR) images before administration during the neonatal period. **C** shows an axial T2-weighted MR image at 18 months old.

The infant in the low-dose cohort exhibits severe diffuse white matter involvement with parenchymal hemorrhages, particularly in the frontal lobes.

D and **E** display axial T1- and T2-weighted MR images before administration during the neonatal period. **F** presents an axial T2-weighted MR image at 18 months old.

The infant in the high-dose cohort displays focal signal abnormalities in the deep white matter of the occipital areas (arrows).
